# Radiofrequency Ablation and Intrauterine Transfusion in a Delayed Diagnosed Acardiac Twin Pregnancy

**DOI:** 10.1155/2023/3243820

**Published:** 2023-08-30

**Authors:** Fatemeh Rahimi-Sharbaf, Mahboobeh Shirazi, Kamran Hessami, Maasoumeh Saleh, Fatemeh Golshahi, Sara Saeedi, Abolfazl Shirdel Abdolmaleki, Seyede Houra Mousavi Vahed, Behnaz Nouri, Behrokh Sahebdel

**Affiliations:** ^1^Department of Obstetrics and Gynecology, Maternal Fetal and Neonatal Research Center, Yas Hospital, Tehran University of Medical Sciences, Tehran, Iran; ^2^Department of Obstetrics and Gynecology, Baylor College of Medicine, Houston, TX, USA; ^3^Department of Obstetrics and Gynecology, Shariati Hospital, Tehran University of Medical Sciences, Tehran, Iran; ^4^Maternal-Fetal Medicine Research Center, Shiraz University of Medical Sciences, Shiraz, Iran; ^5^Department of Obstetrics and Gynecology, Mashhad University of Medical Sciences, Mashhad, Iran; ^6^Department of Community Medicine, School of Medicine, Alborz University of Medical Sciences, Karaj, Iran

## Abstract

Twin reversed arterial perfusion (TRAP) sequence or acardiac twin is a rare and severe complication of monochorionic multiple pregnancies. Acardiac twin accounts for 10% of all TRAP sequences, which is the most morphologically developed acardius. We present an undiagnosed TRAP sequence case up to 24 weeks of gestation who underwent successful amnioreduction, radiofrequency ablation (RFA), and intrauterine transfusion (IUT). During follow-up, hydrops of surviving co-twin disappeared, and fetal heart function improved. Finally, a healthy girl weighing 2400 g was born at 36 weeks of gestation. To our knowledge, this is the first reported acardiac twin pregnancy, which requires IUT, in addition to RFA, due to late diagnosis. Therefore, this case report presents successful management options for TRAP sequence cases diagnosed late in pregnancy.

## 1. Introduction

Current evidence suggests that monozygotic twinning occurs more often after assisted reproductive technologies than spontaneous conception [1, 2]. Monochorionic (MC) twin pregnancies are at increased risk of adverse outcomes because of vascular anastomoses, which connect two fetal circulation systems [1, 3, 4]. Twin reversed arterial perfusion (TRAP) sequence is a rare condition (with a prevalence of 1 in 35,000 overall pregnancies) of MC twin pregnancies, in which one fetus does not have a functional heart and the cardiac system of one of the fetuses performs the work of supplying blood for both fetuses [5, 6]. The twin supplying blood is known as the “pump twin.” The increased demands of the heart of pumping twin puts the fetus at an increased risk of cardiac failure, fetal hydrops, and it can result in a poor perinatal outcome [5, 7]. The “acardiac twin” usually lacks a functional heart associated with poorly developed body parts, such as missing head, limbs, and torso [5, 7].

Despite recent advances in prenatal diagnosis, complications associated with MC multifetal gestations remain unchanged. Management of pregnancies affected by the TRAP sequence poses unresolved challenges to technique choice and optimal timing of intervention [8]. We present a case of acardiac twin pregnancy complicated by fetal hydrops but treated with radiofrequency ablation (RFA) and intrauterine transfusion (IUT) for a hydropic pump twin in the late second trimester.

## 2. Case Presentation

**Figure 1:**
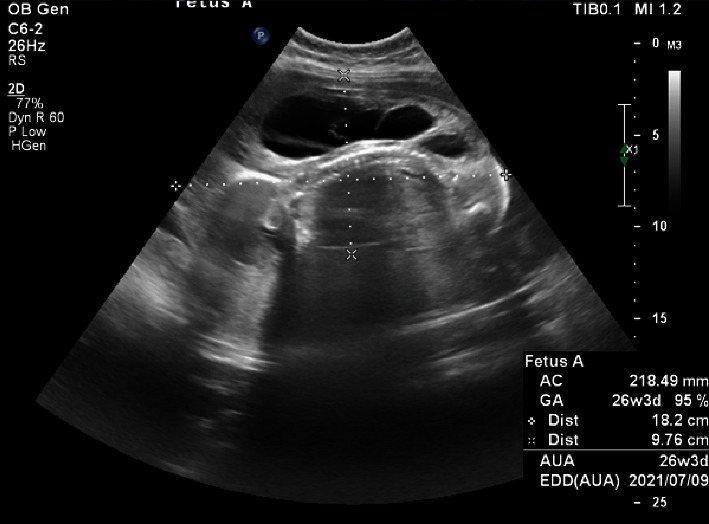
A large mass with generalized edema 18 cm × 9.7 cm in size.

**Figure 2:**
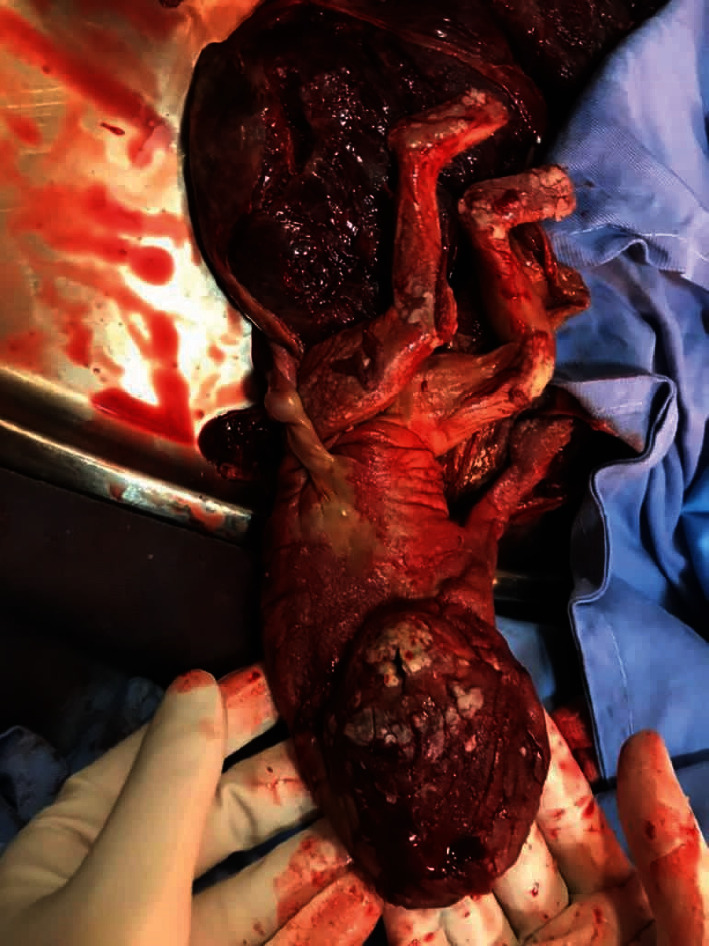
Acardiac twin weighing 230 g after vaginal delivery.

A 27-year-old woman, primigravida, at 23 weeks of gestation was referred to our center due to polyhydramnios. Her pregnancy was spontaneously conceived, and her past medical and drug history were unremarkable. She had two ultrasound reports at 12 and 18 weeks of gestation (nuchal translucency and anomaly scan, respectively), which showed a MC monoamniotic (MCMA) twin pregnancy. In both ultrasound reports, a normal fetus and a dead fetus with generalized edema were reported. An obstetrician visited her at 22 weeks of gestation in terms of shortness of breath, and an ultrasound was requested accordingly because of uterine height mismatch with gestational age. She was referred to our center because of polyhydramnios. An ultrasound at our center showed a large mass with generalized edema 18 cm × 9.7 cm in size (Figure 1) and a fetus with hydrops (subcutaneous edema and pericardial effusion). The mass included the upper and lower parts of the body (head, chest, pelvis, spine, and lower limbs) on detailed examination. The upper limbs were not formed, the skull was fully formed, but brain structures were not correctly formed, the spine and lower extremities were fully formed, and there was no heart, but kidneys were visible. There was a supplying vessel in Doppler examination to that mass, which was a single arterial type with reversal flow. All of this suggested a missed diagnosed acardiac twin pregnancy. Detailed ultrasonographic examination and echocardiography of the pump twin revealed no apparent abnormalities except hydrops fetalis (HF) and heart failure. The ratio of the weight of the acardiac twin to the weight of the pump twin was 0.78. The volume of amniotic fluid (AF) had increased (AF index: 40 cm), and there was evidence of placentomegaly. In the middle cerebral artery Doppler examination, peak systolic velocity was 1.96, indicating fetal anemia. Due to severe polyhydramnios and the mother's dyspnea, amnioreduction was performed first, and about 3 L of AF was drained. A sample of AF was sent for karyotype, which was normal. Amnioreduction also helped make subsequent interventions, including RFA and IUT, easier. A day after the ultrasound, umbilical cord occlusion in the acardiac twin was successfully performed by RFA. Day 1 after RFA, cordocentesis was performed, and 30 mL of packed red blood cell was transfused due to severe fetal anemia [hemoglobin (Hgb): 4 g/dL, reticulocyte count: 40%, and corrected reticulocyte count: 10.6%]. Post IUT, Hgb was 10 g/dL. The patient was discharged 48 hours after IUT and followed up in the outpatient clinic one week after the procedure. Fetal hydrops resolved after one week, and cardiac function improved. After the resolution of fetal hydrops, pregnancy was followed as a singleton pregnancy. Finally, at 36 weeks gestational age, a female newborn weighing 2400 g was born (spontaneous vaginal delivery due to preterm labor) with Apgar scores of 9 and 10 at one and five minutes, respectively. The baby's Arterial Blood Gas showed pH: 7.35, and Hgb was 13.5 g/dL. The weight of acardiac twin was 230 g (Figure 2).

## 3. Discussion

We present an undiagnosed TRAP sequence case up to 24 weeks gestation who underwent successful amnioreduction, RFA, and IUT. To our knowledge, it is the first reported acardiac twin pregnancy, which requires intrauterine blood transfusion in late diagnosis.

There are three ways to perform cord occlusion for acardiac twins and these include: RFA, bipolar cord coagulation, and laser coagulation. In a systematic review by Mone et al. [9], they reported that therapeutic interventions with cord occlusion have better perinatal outcomes than conservative management, especially in pregnancies with one or more poor prognostic features. In most fetal centers, RFA and bipolar cord coagulation are reserved for candidates with TRAP sequence presenting early in pregnancy, such as at least 16 weeks of gestation, but with the intervention preferably performed greater than 18 weeks of gestation, due to concerns about a higher rate of complications, such as preterm prelabor rupture of fetal membranes associated with earlier intervention. Lewi et al. [10] reported that TRAP carries high mortality between the first and early second trimester. In the above-mentioned study, among 24 pregnancies diagnosed in the first trimester, there was the spontaneous demise of pump twin, spontaneous arrest of flow, and persistent flow toward acardiac twin in 33%, 21%, and 26% pregnancies at 16, 17, and 18 weeks, respectively. Tavares de Sousa et al. [11] reported a live birth rate of 92% in 12 TRAP sequence cases following interstitial laser therapy in the first trimester. Hence, intra-fetal laser therapy was suggested to indicate early TRAP presentations, as it can be performed as early as the late first trimester. The clinical situation and preference of the operator are important considerations in the management of the TRAP sequence. The method used in our center to treat the TRAP sequence is RFA. There is a great concern regarding the risk of preterm PROM when performing minimally invasive therapy. Lee et al. [12] and Sugibayashi et al. [13] reported a 17% incidence of preterm PROM after RFA treatment in 98 cases and 2.9% in 40 cases, respectively. MCMA twins or acardiac twins are associated with a high risk of pump twin death after RFA [13].

After umbilical cord occlusion (RFA and/or laser coagulation), 80–90% of pump twins survive with an average gestational age at birth between 35 and 36 weeks. Following successful cord occlusion with subsequent reassuring fetal and maternal surveillance, full-term delivery based on obstetric indications is an appropriate goal.

One or more of the following poor prognostic factors raise the risk of pump twin mortality and this includes pump twin hydrops or high-output heart failure, abnormal pump twin Doppler velocimetry studies, the ratio of the weight of acardiac twin to the weight of the pump twin greater than 0.7, and polyhydramnios [13–15]. Our case had all the above-mentioned risk factors. These factors are all indications for therapeutic interventions. Moreover, we decide to perform IUT to help speed up fetal recovery due to late diagnosis and to reduce worsening complications, such as severe fetal anemia, HF, fetal cardiac dysfunction, polyhydramnios, and stillbirth [16].

In conclusion, regarding the heterogeneity of TRAP sequence and different phenotypes, it is more difficult to diagnose despite being a rare disease. Physicians' awareness of this complication and timely referral of MC twin pregnancies to perinatal specialists is important in early diagnosis and management of the TRAP sequence.

## Data Availability

Data supporting this research article are available from the corresponding author or first author on reasonable request.
